# Interpersonal violence and depression in Brazil: A cross-sectional analysis of the 2019 National Health Survey

**DOI:** 10.1371/journal.pgph.0001207

**Published:** 2022-12-02

**Authors:** Daniel Harding, Charlie F. M. Pitcairn, Daiane Borges Machado, Luis Fernando Silva Castro De Araujo, Christopher Millett, Thomas Hone

**Affiliations:** 1 Department of Primary Care and Public Health, Public Health Policy Evaluation Unity, Imperial College London, London, United Kingdom; 2 Faculty of Public Health and Policy, London School of Hygiene and Tropical Medicine, London, United Kingdom; 3 Centre for Data and Knowledge Integration for Health, Gonçalo Moniz Institute, Oswaldo Cruz Foundation, Salvador, Brazil; 4 Virginia Institute for Psychiatric and Behavioral Genetics, Virginia Commonwealth University, Richmond, Virginia, United States of America; University of Liverpool, UNITED KINGDOM

## Abstract

Depression and interpersonal violence are issues of increasing public health concern globally, especially in low-and-middle income countries. Despite the known relationship between interpersonal violence and an increased risk of depression, there is a need to further characterise the experience of depression in those who have experienced violence, to better develop screening and treatment interventions. A cross-sectional analysis was conducted on responses from the 2019 Brazilian National Health Survey. The prevalence of depression (both clinician-diagnosed, and Patient Health Questionnaire (PHQ-9) screened) were estimated by type of violence experienced in the preceding 12 months (none, physical violence, sexual violence, physical and sexual violence, or threat of violence). Logistic regression models assessed the associations between violence and depression after adjusting for socioeconomic and demographic factors. Of 88,531 respondents, 8.1% experienced any type of violence. Compared to those not experiencing violence, those who experienced any type of violence had a higher prevalence of clinician-diagnosed or PHQ-9-screened depression (e.g. the prevalence of clinician-diagnosed depression was 18.8% for those experiencing sexual violence compared to 9.5% for those not experiencing violence). Both undiagnosed and untreated depression were also more prevalent in those experiencing any type of violence. In logistic regression models, any experience of violence was associated with a higher odds of depression (e.g. aOR = 3.75 (95% CI: 3.06–4.59) for PHQ-9-detected depression). Experiencing violence was also associated with a higher likelihood of having depression which was undiagnosed (e.g. in those who experienced sexual violence: aOR of 3.20, 95% CI 1.81–5.67) or untreated (e.g. in those who experienced physical and sexual violence: aOR = 8.06, 95% CI 3.44–18.9). These findings highlight the need to consider screening for depression in those affected by violence, and to prioritise mental healthcare in communities affected by violence.

## Introduction

Depression is the second-most prevalent mental health disorder globally, affecting an estimated 264 million people in 2017 [1], and is the 13^th^ leading cause of disability-adjusted life years worldwide as of 2019 [2]. Beyond the immediate detriments to quality of life, depression is associated with increased incidence, morbidity, and mortality of numerous physical non-communicable diseases [3], and is the mental health disorder most associated with suicide [4]. Effective treatments exist, with clinical guidelines typically recommending antidepressant medication and/or psychotherapy [5]. Despite this, disparities between those needing and those receiving treatment for depression are a global challenge, with the highest disparities in low- and middle-income countries (LMICs) [6]. Recognising that up to 85% of mental health disorders go untreated in LMICs, the World Health Organization (WHO) has urged countries to prioritise expanding treatment access to comprehensive mental healthcare [7].

Violence is increasingly recognised as a public health problem, and one which disproportionately effects LMICs [8]. The WHO has classified violence along several dimensions, including categorising violent acts as either physical, sexual, psychological, or neglectful, or considering violence as either self-directed, interpersonal, or collective [8]. Any violence, whether witnessed or directly experienced, has been strongly associated with developing depression and suicidal behaviour, in both high-income countries and LMICs [9]. At the municipality-level in Brazil, higher homicide rates have even been associated with higher suicide rates [10]. There is less research assessing whether those with depression and prior experience of violence have a different likelihood of having their depression diagnosed and treated. Several US studies have shown that those with mental health problems who have experienced interpersonal violence (except for women who experienced sexual violence) utilise mental healthcare services at lower rates [11, 12], although measuring service utilisation does not fully reflect rates of diagnosis or treatment.

Research into the links between violence and depression is made more complicated by the plausibly bidirectional relationship between depression and violence [13–15]. Care must also be taken in considering the distinction between issues of healthcare access, and healthcare utilisation, in considering potential explanations for undiagnosed or untreated depression. Further complicating this understanding, is the confounding factor of socioeconomic inequality (measured by either income or education), which is independently associated with depression, poorer access to healthcare, and experiencing violence [16, 17]. Better understanding of these interactions is vital to inform health policy and service provision.

Brazil is a compelling environment to study violence and depression. Within South America, Brazil has the second highest homicide rate (a common proxy measure for violence generally) at 30.5 deaths per 100,000 (2017 data) [18, 19]. Brazil is the 16^th^ most socioeconomically unequal country in the world, measured by Gini index (2020 data) [20]. Furthermore, in 2015 Brazil was estimated to have the 5^th^ highest prevalence of adult depression in the world [21]. Mental healthcare in Brazil has undergone significant development in the last three decades. Since 1988, universal free mental healthcare has been available under the Unified Health System (SUS), although one third of Brazilians utilise private healthcare [22]. The number of inpatient psychiatric beds has continually been below government targets (0.12 beds per 1,000 people in 2016 vs. a target of 0.45), however, there has been large upscaling of community mental healthcare, with the number of Centres of Psychosocial Care multiplying by over sevenfold, between 2004 and 2018 [22]. Studies show however that utilisation of mental healthcare in Brazil, is still affected by socioeconomic inequality, with higher use in those with greater education, and lower use in areas with higher homicide rates [23, 24].

This study assesses responses to the 2019 *Pesquisa Nacional de Saúde* (PNS) (National Health Survey), a nationally representative survey conducted in association with the Brazilian Ministry of Health. It describes the association between an experience of violence within the last 12 months, and several depression diagnostic and treatment outcomes. Secondly, it examines the association between experiencing violence and depression treatment outcomes and severity markers, in a subgroup of people who had been diagnosed with depression by a clinician.

## Materials and methods

### Study design and data source

This study is an analysis of cross-sectional data of responses to the 2019 PNS, a survey conducted by the Brazilian Institute of Geography and Statistics (IBGE) in collaboration with the Ministry of Health. The PNS used probabilistic sampling, and collected survey responses from 94,114 residents aged 15 or more, in private households across Brazil, using computer-assisted personal interviews. Regarding participant selection, first, primary sampling units (based on census tracts) were selected with probability proportional to size, defined by the number of permanent private dwellings. Secondly, households were randomly selected from the National Register of Addresses for Statistical Purposes. Thirdly, one resident aged 15 years or older was randomly selected from each household. Responses were weighted for representativeness and to account for non-response. Out of a total of 94,114 participants, 88,531 completed the survey questions on depression and violence, and were included in the analyses. Participants with incomplete responses were excluded (n = 5,583). Surveys were conducted between August 2019 and March 2020. Further information about PNS design is published elsewhere [25].

### Variables

The main variable of interest was whether an individual reported an experience of any violence in the preceding 12 months (full questions provided in S1 Table). As a secondary variable of interest, any experience of violence was subcategorised by the type of violence experienced in the preceding 12 months: either ‘physical violence only,’ ‘sexual violence only,’ ‘both physical and sexual violence,’ or ‘a threat of violence only.’

Outcome variables were related to depression. The first set of outcomes considered a diagnosis of depression: i) a history of clinician-diagnosed depression; and ii) current depression based on calculation of Patient Health Questionnaire (PHQ-9) scores for each respondent. The PHQ-9 is an internationally recognised screening questionnaire for depression based on symptomatology, designed for the outpatient setting, and in this study a score of ≥10 out of 27 was considered to detect current depression [26–28]; iii) current severe depression, defined as having a PHQ-9 score of ≥20 [29]; iv) current undiagnosed depression, defined as having a PHQ-9 score of ≥10, and not having prior clinician-diagnosed depression; v) untreated depression, defined as having a PHQ-9 score of ≥10, and not currently using antidepressant medication or attending psychotherapy.

A second set of outcomes were generated only for a subgroup of respondents previously diagnosed with depression by a clinician (n = 8,242), to further characterise depression severity and treatment. These were (specifically in relation to depression): i) current medication and/or current psychotherapy use; ii) current alternative medicine use; iii) regular clinician follow-up; iv) specialist referral; v) non-attendance of specialist appointment; vi) severe limitation on activities of daily living (ADL); and vii) emergency room attendance in last year.

The following socioeconomic and demographic factors were used in regression models: sex (male or female); age (grouped into 15–24, 25–34, 35–44, 45–54, 55–64, 65–74, ≥75); race/ethnicity (White, Black, Mixed, or other (Asian, Indigenous, or not declared)); region (North, Northeast, Central-West, Southeast, South); area (urban or rural); education (no education or incomplete elementary school, elementary school or incomplete high school, high school or incomplete higher education, or graduate); monthly income as a proportion of the national minimum wage (MW) (grouped as ≤0.5xMW, 0.51-1xMW, 1.01-2xMW, and >2xMW); employment (employed or unemployed); possessing private health insurance; and marital status (married, separated or widowed, and single).

### Statistical analysis

Firstly, prevalence estimates of any experience of violence, and specific types of violence, were estimated for the whole study population (n = 88,531). Survey weights were applied to obtain nationally representative estimates with 95% confidence intervals (95% CI). Secondly, the prevalence of depression outcomes was estimated by different groups experiencing violence. Thirdly, the prevalence of depression treatments and characteristics, of those with clinician-diagnosed depression (n = 8,242), were estimated for different groups experiencing violence.

Logistic regressions were performed to assess the relationship between violence and depression after adjusting for socioeconomic and demographic variables. All models were adjusted for: sex, age, race/ethnicity, region, area, education, monthly income, and marital status. In the first set of logistic regression models, the associations between each depression outcome and an experience of violence (by type) were assessed analysing the whole study sample. A second set of regression models used only the subgroup of respondents who had a previous clinician-diagnosis of depression. Depression treatment and characteristics outcomes were modelled in separate models. Due to the smaller number of respondents, only any experience (or not) of violence was included as the variable of interest, as when subcategorised by type of violence, each outcome was too rare to conduct logistic regression models.

All statistical analysis was conducted using Stata 15 software (Stata Corp., TX, USA), using nationally representative weightings determined by IBGE.

### Ethics statement

Our study was exempted from needing approval by our institutional ethics review board as it uses secondary data that is in the public domain. Ethical approval for the National Survey of Health (PNS 2019) was approved by the Brazilian National Health Ethics Committee (CONEP) (process n° 3.529.376).

## Results

### Prevalence of experiencing violence

The responses from 88,531 individuals (response rate 94.1%) were analysed (Table 1). Overall, 8.1% (95% CI 7.7–8.5%) of respondents reported experiencing any violence in the last 12 months. The rates of experiencing violence were higher for: younger age groups (14.1% (95% CI 12.9–15.5%) in those 15 to 24 years old, vs. 2.5% (95% CI 1.9–3.3%) in those 75 or older); those of Black and mixed race/ethnicity (10.3% (95% CI 9.2–11.4%) and 9.0% (95% CI 8.5–9.6%) respectively); those living in urban areas (8.3%, 95% CI 7.9–8.3%); those with only elementary or incomplete high school level education (10.1%, 95% CI 9.2–11.2%); those without private health insurance (8.7%, 95% CI 8.3–9.2%); and single people (10.7%, 95% CI 10.2–11.4%). There was a decreasing experience of violence with increasing monthly income (10.9% (95% CI 10.1–11.8%) in ≤0.5xMW, vs. 6.1% (95% CI 5.5–6.9%) in >2xMW).

**Table 1:** Characteristics of survey respondents by experience of violence.

	Those who experienced any violence within the last year			Those who did not experience any violence within the last year		
	N	Prevalence (%)	95% CI (%)	N	Prevalence (%)	95% CI (%)
**Sex**						
**Male**	2,985	7.5	7.0–8.0	38,677	92.5	92.0–93.0
**Female**	3,971	8.6	8.1–9.1	42,898	91.4	90.9–91.9
**Age**						
**15–24**	1,144	14.1	12.9–15.5	7,001	85.9	84.5–87.2
**25–34**	1,655	9.3	8.6–10.1	14,315	90.7	89.9–91.4
**35–44**	1,569	8.7	8.0–9.6	16,464	91.3	90.4–92.0
**45–54**	1,215	7.3	6.6–8.2	14,670	92.7	91.9–93.4
**55–64**	827	5.6	5.0–6.2	13,745	94.4	93.8–95.0
**65–74**	406	3.9	3.4–4.6	9,559	96.1	95.4–96.6
**75+**	140	2.5	1.9–3.3	5,821	97.5	96.7–98.1
**Race/ethnicity**						
**White**	2,129	6.6	6.1–7.1	30,280	93.4	92.9–93.9
**Black**	972	10.3	9.2–11.4	9,160	89.8	88.6–90.8
**Mixed**	3,739	9.0	8.5–9.6	40,907	91.0	90.4–91.5
**Asian or Indigenous**	116	5.8	4.2–7.8	1,228	94.2	92.2–95.8
**Region**	** **					
**North**	1,418	9.0	8.2–9.9	15,519	91.0	90.1–91.8
**Northeast**	2,538	8.8	8.3–9.4	28,164	91.2	90.6–91.7
**Central-West**	815	8.0	7.2–8.8	9,366	92.0	91.2–92.8
**Southeast**	1,460	7.8	7.1–8.6	17,975	92.2	91.4–92.9
**South**	725	6.9	6.3–7.6	10,551	93.1	92.4–93.7
**Area**						
**Urban**	5,678	8.3	7.9–8.7	62,542	91.7	91.3–92.1
**Rural**	1,278	6.6	6.0–7.2	19,033	93.4	92.8–94
**Education**						
**None/Incomplete Elementary**	2,436	7.6	7.0–8.2	33,136	92.5	91.8–93.0
**Elementary/Incomplete High**	1,192	10.1	9.2–11.2	10,813	89.9	88.8–90.9
**High/Incomplete Higher**	2,391	8.5	7.9–9.1	24,946	91.5	90.9–92.1
**Graduate**	937	6.3	5.6–7.2	12,680	93.7	92.9–94.4
**Monthly Income**						
**Up to 0.5x MW**	2,239	10.9	10.1–11.8	20,299	89.1	88.2–89.9
**0.51-1x MW**	2,055	8.5	7.9–9.1	23,703	91.5	90.9–92.1
**1.01-2x MW**	1,568	6.8	6.1–7.5	20,592	93.2	92.5–93.9
**>2x MW**	1,093	6.1	5.5–6.9	16,960	93.9	93.1–94.5
**Employment**						
**Employed**	5,044	9.0	8.5–9.5	51,168	91.0	90.5–91.5
**Unemployed**	1,912	6.2	5.8–6.7	30,407	93.8	93.2–94.2
**Private Health Insurance**						
**Yes**	1,211	6.2	5.6–6.9	19,569	93.8	93.1–94.4
**No**	5,745	8.7	8.3–9.2	65,125	91.3	90.8–91.7
**Marital Status**						
**Married**	1,932	5.7	5.1–6.3	33,178	94.4	93.7–94.9
**Separated or widowed**	1,065	7.6	6.9–8.3	14,076	92.4	91.7–93.1
**Single**	3,959	10.7	10.2–11.4	34,321	89.3	88.7–89.9
						
**Total**	6,956	8.1	7.7–8.5	81,575	91.9	91.5–92.3

Prevalence estimates provided generated using nationally representative survey weightings. MW: Minimum Wage. CI: Confidence Intervals.

The estimated prevalence of experiencing violence varied by the type of violence (Table 2), with 3.8% of respondents (95% CI 3.6–4.1%) experiencing only physical violence, 3.5% (95% CI 3.2–3.7%) experiencing only threats of violence, 0.5% (95% CI 0.4–0.6%) experiencing only sexual violence, and 0.3% (95% CI 0.3–0.4%) experiencing both physical and sexual violence. Notably, experiences of sexual violence were markedly more prevalent in females than males (0.7% (95% CI 0.5–0.9%) vs. 0.3% (95% CI 0.2–0.4%) for sexual violence only, and 0.4% (95% CI 0.3–0.5%) vs. 0.2% (95% CI 0.1–0.3%) for both physical and sexual violence).

**Table 2:** Characteristics of survey respondents by types of violence experienced.

	Experience of only physical violence			Experience of only sexual violence			Experience of physical and sexual violence			Experience of only a threat of violence		
	N	Prevalence (%)	95% CI (%)	N	Prevalence (%)	95% CI (%)	N	Prevalence (%)	95% CI (%)	N	Prevalence (%)	95% CI (%)
**Sex**												
**Male**	1,509	3.9	3.5–4.2	126	0.25	0.2–0.4	63	0.2	0.1–0.3	1,287	3.2	2.8–3.5
**Female**	1,656	3.8	3.5–4.3	271	0.65	0.5–0.9	212	0.4	0.3–0.5	1,832	3.7	3.4–4.0
**Age **												
**15–24**	618	7.8	6.9–8.9	95	1.1	0.8–1.5	61	0.7	0.5–1.0	370	4.5	3.7–5.4
**25–34**	802	4.8	4.2–5.5	102	0.4	0.3–0.5	94	0.6	0.4–0.8	657	3.5	3.1–4.0
**35–44**	723	4.0	3.5–4.5	69	0.4	0.3–0.6	54	0.2	0.1–0.3	723	4.1	3.6–4.8
**45–54**	485	3.0	2.5–3.6	62	0.6	0.3–1.4	50	0.2	0.1–0.3	618	3.5	3.1–4.0
**55–64**	335	2.3	2.0–2.7	41	0.2	0.1–0.3	14	0.1	0.0–0.3	437	2.9	2.5–3.4
**65–74**	146	1.5	1.1–1.9	21	0.1	0.1–0.3	2	0.0	0.0–0.1	237	2.3	1.9–2.8
**75+**	56	1.1	0.7–1.7	7	0.1	0.1–0.3	0	-	-	77	1.3	0.9–1.9
**Race/ethnicity**												
**White**	946	3.0	2.7–3.4	136	0.6	0.4–0.8	68	0.2	0.1–0.3	979	2.9	2.6–3.2
**Black**	436	4.9	4.2–5.7	58	0.6	0.4–0.9	35	0.3	0.2–0.6	443	4.4	3.8–5.2
**Mixed**	1,736	4.5	4.1–4.9	197	0.3	0.3–0.4	167	0.4	0.3–0.6	1,639	3.8	3.5–4.2
**Asian or Indigenous**	47	1.8	1.2–2.8	6	0.4	0.1–1.3	5	0.2	0.1–0.6	58	3.3	2.1–5.3
**Region**												
**North**	649	4.4	3.8–5.0	79	0.5	0.4–0,7	58	0.4	0.2–0.5	632	3.8	3.3–4.3
**Northeast**	1,114	4.1	3.7–4.5	157	0.5	0.4–0.7	114	0.4	0.3–0.5	1,153	3.8	3.5–4.2
**Central-West**	366	3.7	3.1–4.3	47	0.5	0.3–0.8	39	0.3	0.2–0.5	363	3.5	3.0–4.0
**Southeast**	679	3.7	3.2–4.3	77	0.5	0.3–0.8	43	0.3	0.2–0.4	661	3.4	3.0–3.9
**South**	357	3.6	3.2–4.2	37	0.3	0.2–0.4	21	0.2	0.1–0.4	310	2.8	2.4–3.2
**Area**												
**Urban**	2,608	4.0	3.7–4.3	316	0.5	0.4–0.6	225	0.3	0.2–0.4	2,529	3.6	3.3–3.8
**Rural**	557	3.1	2.6–3.6	81	0.4	0.3–0.6	50	0.3	0.2–0.5	590	2.7	2.4–3.2
**Education**												
**None/Incomplete Elementary**	1,120	3.6	3.2–4.0	116	0.4	0.2–0.7	77	0.3	0.2–0.4	1,123	3.3	3.0–3.7
**Elementary/Incomplete High**	615	5.8	5.0–6.7	57	0.5	0.3–0.9	58	0.4	0.3–0.7	462	3.4	3.0–4.0
**High/Incomplete Higher**	1,053	3.9	3.5–4.3	153	0.6	0.4–0.7	103	0.3	0.2–0.5	1,082	3.7	3.3–4.2
**Graduate**	377	2.6	2.1–3.3	71	0.4	0.2–0.5	37	0.2	0.1–0.4	452	3.1	2.7–3.7
**Monthly Income**												
**Up to 0.5x MW**	1,073	5.3	4.8–5.9	111	0.7	0.4–1.3	113	0.6	0.4–0.8	942	4.4	3.9–4.9
**0.51-1x MW**	921	4.1	3.7–4.6	110	0.4	0.3–0.5	72	0.3	0.2–0.4	952	3.7	3.4–4.1
**1.01-2x MW**	681	3.1	2.7–3.6	101	0.4	0.3–0.6	53	0.2	0.2–0.4	733	3.1	2.7–3.5
**>2x MW**	490	2.9	2.4–3.5	75	0.4	0.3–0.6	37	0.2	0.1–0.3	491	2.6	2.3–3.1
**Employment**												
**Employed**	2,325	4.3	4.0–4.6	297	0.5	0.4–0.7	206	0.3	0.3–0.4	2,216	3.8	3.6–4.1
**Unemployed**	840	3.0	2.6–3.5	100	0.3	0.2–0.4	69	0.2	0.2–0.3	903	2.7	2.4–3.0
**Private Health Insurance**												
**Yes**	533	2.7	2.4–3.2	85	0.3	0.2–0.5	43	0.2	0.0–0.3	550	3.0	2.5–3.5
**No**	2,632	4.3	3.9–4.6	312	0.5	0.4–0.7	232	0.4	0.3–0.4	2,569	3.6	3.4–3.9
**Marital Status**												
**Married**	804	2.4	2.1–2.8	77	0.2	0.1–0.3	44	0.1	0.1–0.2	1,007	2.9	2.6–3.3
**Separated or widowed**	433	3.2	2.8–3.8	59	0.4	0.3–0.6	36	0.3	0.1–0.5	537	3.7	3.2–4.2
**Single**	1,928	5.5	5.1–6.0	261	0.8	0.6–1.0	195	0.5	0.4–0.7	1,575	3.9	3.6–4.3
												
**Total**	3,165	3.8	3.6–4.1	397	0.5	0.4–0.6	275	0.3	0.3–0.4	3,119	3.5	3.2–3.7

Prevalence estimates provided generated using nationally representative survey weightings. MW: Minimum Wage. CI: Confidence Intervals.

### Depression and experiencing violence

**Fig 1:**
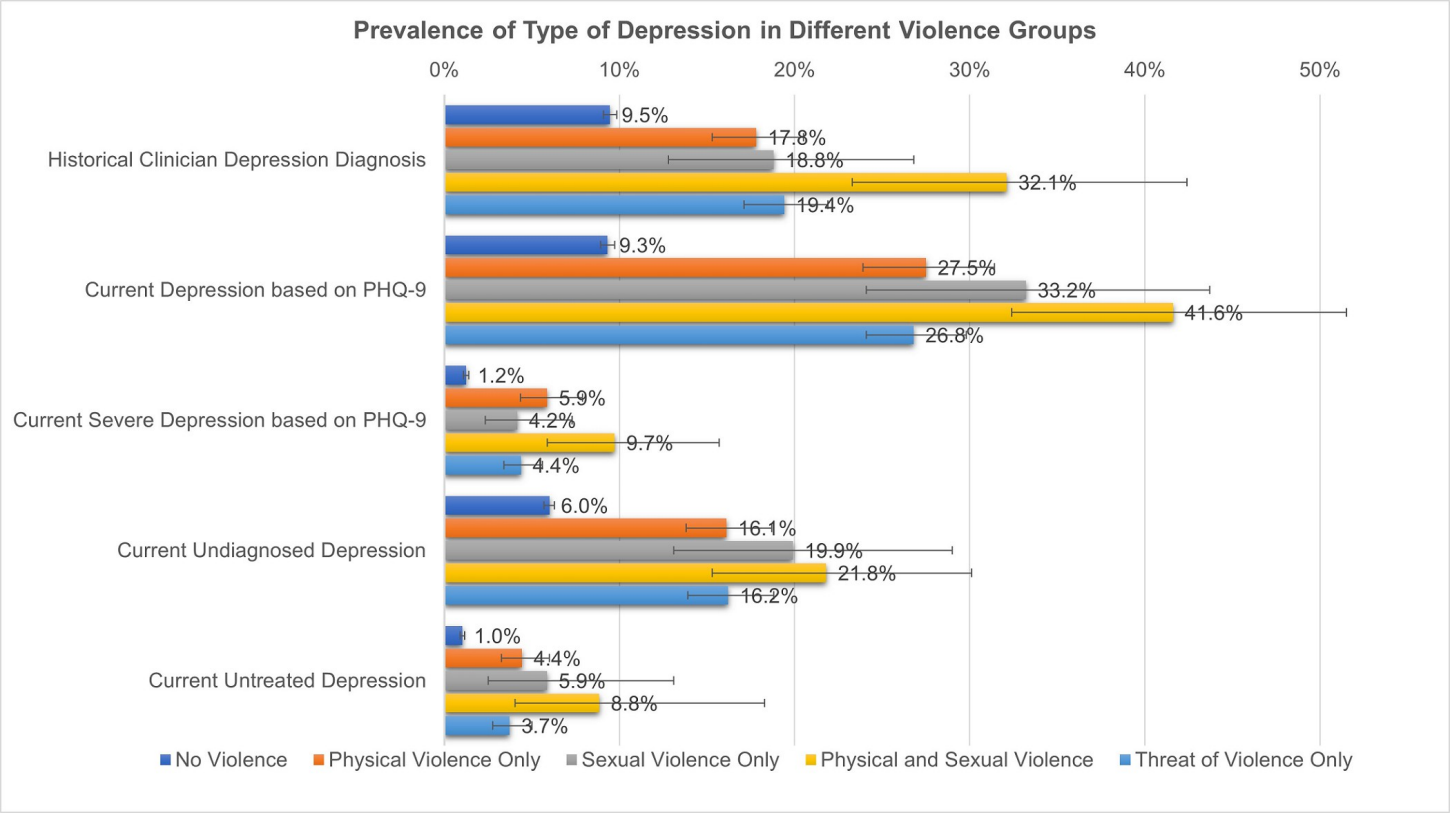
Prevalence of different types of depression across populations experiencing violence. Error bars represent 95% confidence intervals. PHQ-9: Patient Health Questionnaire.

More than a quarter of those who experienced any form of violence had PHQ-9-detected depression (Fig 1, S2 Table). Compared to those who did not experience violence, the prevalence of PHQ-9-detected depression was higher in those who had experienced violence. This pattern was across all forms of violence, clinician- or PHQ-9-deteceted, as well as severe depression, undiagnosed depression, and untreated depression. For example, the prevalence of undiagnosed depression for those who had experienced physical violence was 16.1% (95% CI 13.8–18.7%) compared to 6.0% (95% CI 5.7–6.3%) for those who hadn’t experience violence. The rate of untreated depression was four time higher for those who experienced physical violence (4.4%, 95% CI 3.3–6.0%) compared to those who had no experience of violence (1.0%, 95% CI 0.9–1.2%). The prevalence of PHQ-9-screened depression in those who had experienced sexual violence was 33.2% (95% CI 24.1–43.7%), compared to 9.3% (95% CI 8.9–9.7%) in those who hadn’t experienced violence. Those who had experienced sexual violence were also twice as likely to have been diagnosed with depression by a clinician (18.8%, 95% CI 12.8–26.8%), compared to those who hadn’t experienced violence (9.5%, 95% CI 9.1–9.9%).

Those who had experienced both physical and sexual violence, had the highest estimated prevalence of every type of depression. For example, the prevalence of current severe depression in this group was 9.7% (95% CI 5.9–15.7%), compared to 1.2% (95% CI 1.1–1.4%) in those who hadn’t experienced violence.

### Depression treatment in those who experienced violence

A subgroup analysis was conducted on those previously diagnosed with depression by a clinician (n = 8,242) (Table 3). Overall, those who experienced violence had higher utilisation of therapy in comparison to those without an experience of violence. In those who had experienced sexual violence, 35.8% (95% CI 22.1–52.1%) were currently undergoing psychotherapy for depression, compared to 17.9% (95% CI 16.5–19.5%) in those who hadn’t experienced violence. The prevalence of specialist referral for depression in those who had experienced sexual violence was 92.9% (95% CI 80.3–97.7%), which was comparatively higher than in those with no experience of violence (74.0%, 95% CI 71.7–76.2%). Those who experienced sexual violence, most reported severe limitation on ADL due to depression, with a prevalence of 28.8% (95% CI 16.9–44.6%), compared to 11.1% (95% CI 9.9–12.5%) in those without experience of violence.

**Table 3:** Prevalence of depression treatments and characteristics by types of violence experienced, in those with previous clinician-diagnosed depression (n = 8,242).

	No experience of violence			Experience of only physical violence			Experience of only sexual violence			Experience of physical and sexual violence			Experience of only a threat of violence		
	N	Prevalence (%)	95% CI (%)	N	Prevalence (%)	95% CI (%)	N	Prevalence (%)	95% CI (%)	N	Prevalence (%)	95% CI (%)	N	Prevalence (%)	95% CI (%)
**Current Medication for Depression**	3,707	54.8	52.8–56.8	240	45.8	37.2–54.7	45	45.2	29.5–62.0	40	41.8	26.0–59.5	277	48.5	41.6–55.5
**Current Therapy for Depression**	1,247	17.9	16.5–19.5	127	26.5	18.8–35.9	29	35.8	22.1–52.2	24	30.1	16.3–48.8	125	20.5	15.5–26.6
**Current Medication and Therapy for Depression**	1,034	14.9	13.5–16.3	96	16.1	12.2–21.0	20	23.7	13.1–38.9	15	19.7	8.61–39.0	102	15.7	11.3–21.4
**Current Alternative Medicine for Depression**	389	5.7	4.8–6.7	37	10.3	4.1–23.5	7	7.8	3.1–18.3	7	5.9	2.4–13.7	40	7.8	4.5–13.3
**Regular Follow-up for Depression**	2,512	37.6	35.6–39.7	194	44.6	35.3–54.3	31	34.6	21.2–51.0	39	38.1	23.1–55.8	211	36.8	30.3–43.7
**Specialist Referral for Depression**	3,229	74.0	71.7–76.2	288	78.6	69.6–85.5	55	92.9	80.3–97.7	58	89.5	76.8–95.6	289	75.9	68.3–82.2
**Non-attendance of Specialist Appointment**	247	15.0	12.5–17.9	35	28.5	13.1–51.1	6	16.1	4.8–42.4	8	7.9	3.1–18.5	36	22.1	12.5–36.1
**Severe Limitation on ADL due to Depression**	775	11.1	9.88–12.5	90	18.9	12.4–27.6	21	28.8	16.9–44.6	20	18.4	9.8–31.9	103	15.2	11.3–20.1
**Emergency Room Attendance due to Depression in Last Year**	169	0.3	0.2–0.3	21	0.7	0.3–1.4	1	0.1	0.0–0.8	5	2.3	0.6–7.7	9	0.4	0.2–1.0

ADL: Activities of Daily Living. CI: Confidence Intervals.

### Regression models on depression and violence

Logistic regression models were used to assess the associations between depression and violence whilst adjusting for socioeconomic factors (Table 4). In separate models for each type of depression diagnosis, an experience of any type of violence was associated with significantly higher odds of depression. Individuals who experienced physical violence or a threat of violence were 2.5 times more likely to have clinician-diagnosed depression (aOR = 2.54, 95% CI 2.11–3.06 and aOR = 2.50, 95% CI 2.13–2.93 respectively), and 3.5 times more likely to have PHQ-9-screened depression (aOR = 3.75, 95% CI 3.06–4.59 and aOR = 3.49, 95% CI 3.00–4.07 respectively). Combined physical and sexual violence had the highest association with current severe depression (aOR = 7.79, 95% CI 4.21–14.4). All types of violence were significantly associated with higher odds of having undiagnosed depression, and untreated depression. For instance, experience of sexual violence in comparison to no experience of violence, had an aOR of 3.20 (95% CI 1.81–5.67) for undiagnosed depression, and 4.60 (95% CI 1.79–11.6) for untreated depression. Combined physical and sexual violence had the highest association with untreated depression (aOR = 8.06, 95% CI 3.44–18.9). Full output of these models is provided in S3 Table.

**Table 4:** Results from logistic regression models assessing associations between depression measures and types of violence experienced.

		Clinician-diagnosed Depression	Current PHQ-9-deteceted Depression	Current Severe Depression based on PHQ-9	Current Undiagnosed Depression	Current Untreated Depression
**No Violence**	aOR	1 (Ref)	1 (Ref)	1 (Ref)	1 (Ref)	1 (Ref)
**Physical Violence Only**	aOR	2.54**	3.75**	4.83**	2.84**	4.31**
	95%CI	2.11–3.06	3.06–4.59	3.48–6.70	2.32–3.47	3.01–6.19
**Sexual Violence Only**	aOR	2.06*	4.04**	2.70*	3.20**	4.60**
	95%CI	1.13–3.76	2.27–7.21	1.21–6.00	1.81–5.67	1.79–11.6
**Physical & Sexual Violence**	aOR	5.86**	6.39**	7.79**	3.65**	8.06**
	95%CI	3.72–9.23	4.21–9.70	4.21–14.4	2.35–5.67	3.44–18.9
**Threat of Violence Only**	aOR	2.50**	3.49**	3.39**	2.85**	3.43**
	95%CI	2.13–2.93	3.00–4.07	2.55–4.52	2.36–3.45	2.46–4.79

Logistic regression models investigating the association between experience of violence and various depression variables in whole survey population. Other variables included in the model are: gender; age; race/ethnicity; region; urban or rural locality; household income group; highest educational attainment; and marital status. Statistical significance of the adjusted Odd’s Ratio (aOR) is indicated by * for p<0.05, or ** for p<0.01. aOR confidence intervals (CI) are reported at the level of 95%. PHQ-9: Patient Health Questionnaire.

### Regression models on depression treatments and characteristics, and violence

Logistic regression models were carried out for the subgroup of 8,242 respondents who had clinician-diagnosed depression and had answered questions on treatment and severity (Table 5). An experience of violence was not significantly associated with specific depression treatments, such as antidepressants or therapy, although there was a non-significant trend of those who experienced violence being more likely to attend therapy for depression (aOR = 1.30, 95% CI 1.00–1.69). An experience of violence was significantly associated with an increased likelihood of failing to attend specialist appointments (aOR = 1.79, 95% CI 1.02–3.15), as well as two markers of depression severity: severe limitation on ADL (aOR = 1.61, 95% CI 1.29–2.01); and emergency room attendance in the last year due to depression (aOR = 2.09, 95% CI 1.21–3.61).

**Table 5:** Results from logistic regression models assessing associations between depression treatments and characteristics, and experiencing violence.

		Current Medication for Depression	Current Therapy for Depression	Current Medication and Therapy for Depression	Current Alternative Medicine for Depression	Regular Follow-up for Depression	Specialist Referral for Depression	Non-attendance of Specialist Appointment	Severe Limitation on ADL due to Depression	Emergency Room Attendance due to Depression in Last Year
**No Violence**	**aOR**	1	1	1	1	1	1	1	1	1
**Violence**	**aOR**	0.81	1.3	1.03	1.68	1.05	1.17	1.79*	1.61**	2.09**
	**CI**	0.65–1.01	1.00–1.69	0.79–1.35	0.92–3.05	0.83–1.33	0.86–1.59	1.02–3.15	1.29–2.01	1.21–3.61

Logistic regression models investigating the association between experience of violence within the last 12 months, and various depression variables, in a subgroup of survey participants who had previously been diagnosed with depression by a clinician (n = 8,242). Other variables included in the model are: gender; age; race/ethnicity; region; urban or rural locality; household income group; highest educational attainment; and marital status. Statistical significance of the adjusted Odd’s Ratio (aOR) is indicated by * for p<0.05, or ** for p<0.01. aOR confidence intervals (CI) are reported at the level of 95%. ADL: Activities of Daily Living. PHQ-9: Patient Health Questionnaire.

## Discussion

In 2019, an estimated 10.8% of adults had depression in Brazil, whilst 8.1% had experienced any form of violence in the last 12 months. Regardless of the type, individuals who experienced violence had a significantly higher prevalence of depression (either by PHQ-9 screening or historic clinician-diagnosis), than those who had not experienced violence. Additionally, those who experienced violence, had higher rates of undiagnosed and/or untreated depression. After adjustment for socioeconomic and demographic factors, these associations persisted. For instance, those who had experience of physical violence were 3.75 times as likely to have PHQ-9-screened depression as those with no experience of violence, and those with experience of sexual violence, were 3.20 times as likely to have undiagnosed depression.

These results are concordant with existing studies. Witnessing or directly experiencing violence has been associated with depression diagnosis, and severity of depression, in two large Brazilian cities [18, 30]. Another study from Brazil, of women experiencing intimate partner violence (IPV), also demonstrated a significant association between mental health disorders and experience of violence [31]. Compared to these prior Brazilian studies, our study utilises a larger sample, is nationally rather than regionally representative, and explores depression which is untreated or undiagnosed. The association between IPV and depression more generally has been similarly demonstrated in other LMICs [32, 33].

Given the nature of our cross-sectional analysis, our results do not establish causality, or the direction of relationship between experiencing violence and depression, or if a relationship is unidirectional or bidirectional. There are however numerous plausible reasons for why experiencing violence could be associated with development of depression. Violence can trigger depression onset, and continued violence, particularly within an abusive relationship, can have deep effects on self-esteem, personality, cognition, and resilience to other stressors [34]. Being a victim of an act of violence, can also induce repetitive negative emotions such as distress, self-blame, denial, shame, and confusion [35]. Beyond this, there can be persistent mood alteration, and behavioural changes which contributes to a depressed state, such as social withdrawal or substance misuse [36]. Impaired psychological resilience has been proposed as an important factor in explaining why certain individuals develop psychopathology after traumatic experiences [37]. Of relevance to explaining our findings, decreased resilience has been associated with early life experience of interpersonal violence [38], and this early experience has been associated with depression in later life [39]. Conversely, it is possible that those who already experience depression are at higher risk of experiencing violence in the future, as has been demonstrated in young women experiencing IPV [13, 14], and crime victimisation in people with severe mental health disorders [40].

Our study found that those who experienced both sexual and physical violence, as opposed to either type individually, had comparatively higher rates of depression, and more severe depression. This is similar to a study of IPV in Bangladesh, which demonstrated that multiple types of violence experienced in combination (or increasing frequency), was related to an increasingly higher risk ratio of having depression [41]. IPV appears to be the most predominantly studied type of interpersonal violence in relation to adult depression in LMICs, and overall, there has been comparatively less study of other forms of violence [42]. Interpersonal violence between community members, has been shown to be associated with depression in post-conflict communities [43, 44], and described in the aftermath of political violence [45]. Our findings suggest the association between violence and depression extends beyond IPV and is generalisable to interpersonal violence in an adult population not experiencing conflict.

Our analysis showed that sexual violence was predominantly experienced by women, with 2.6 times the estimated prevalence compared to men, and 2 times the estimated prevalence of experience of combined sexual and physical violence (a category of violence we repeatedly found to have the highest odds ratio for severe, undiagnosed, and untreated depression). We also found that women were 2.2 times as likely to have undiagnosed depression as men, and 2.7 times as likely to have untreated depression (S3 Table). It is well established that both sexual violence, and IPV, are predominantly experienced by women [8, 46]. Given these findings, it would be highly valuable to further investigate this group. Firstly, this could aid more effective identification and treatment of depression in women in Brazil who have experienced sexual violence or IPV, alongside optimisation of services to better address the unmet mental healthcare needs of this important group. Secondly, this could further support wider social policy aiming to eliminate IPV and sexual violence.

Our analysis found a highly similar strength of association between directly experiencing physical violence and current depression, and experiencing only threatened violence and current depression. This suggests that living with the anticipation of threatened violence may have a similarly significant psychological impact to actual enacted violence.

Undiagnosed or untreated depression was significantly associated with personal experience of violence, however our survey lacked measures of healthcare-seeking behaviour to better understand this association. Elsewhere, in areas with higher homicide rates those with depression have been shown to be less likely to access help from mental health services, due to a combination of personal (e.g. personal health beliefs), cultural (e.g. normalisation of traumatic experiences), and service-level (e.g. health professionals preferring to work in safer areas) factors [23]. Lack of treatment could potentially partially explain why we found experience of violence was associated with markers of severer depression. Of those who experienced violence, 16.1–21.8% had undiagnosed depression (based on PHQ-9-screening), which was higher than the rates of untreated current depression (3.7–8.8%). In addition to recall bias, one possible explanation for this discrepancy may be that those with undiagnosed depression are accessing mental health treatments without clinician-diagnosis, or are receiving antidepressant medication or psychotherapy following a co-morbid mental health diagnosis, such as generalised anxiety disorder [47].

Amongst those who had received a clinician-diagnosis of depression, those who had experienced violence were more frequently treated with psychotherapy, in comparison to those without experience of violence. Randomised-control trials in LMICs have shown those who experienced IPV had significantly more benefit from psychotherapy than those who had not experienced IPV, even if therapy didn’t specifically address IPV-related experiences [48]. Use of regular follow-up and referral to specialists appeared similar across categories of violence, except for those who had experienced sexual violence (singularly or in combination with physical violence), who were most frequently referred to specialists. This is suggestive of a higher degree of clinician concern and/or perceived severity of symptoms, when treating those with depression who had experienced sexual violence.

Our study has several limitations, including that the associations identified here do not demonstrate causality. It was not possible to fully determine if depression occurred after experiencing violence, although the PHQ-9 screening assessed current symptoms at the interview. There is also a risk of recall and non-response bias, which may have reduced reporting of experiences of violence. Another limitation is that the questionnaires were conducted in the home [25]. Although trained interviewers endeavoured to ensure confidentiality, it is conceivable that interviews in this setting may have inhibited reporting of IPV or sexual violence, underestimating the prevalence of these types of violence. This limitation is of particular concern in estimating the true prevalence of sexual violence experienced by women, where stigma or safety concerns may have prohibited disclosure, as is believed to occur in similar studies [49]. Finally, we cannot determine from our study to what extent undiagnosed or untreated depression results from lack of healthcare access, or problems with healthcare utilisation. Prior research has demonstrated that utilisation is affected by individual and community factors. One study showed lower rates of doctor consultation in areas of high violence in Sao Paulo [50], whilst qualitative research in a favela in Rio de Janeiro with a high level of community violence, identified that despite access, numerous women with mental health concerns struggled to utilise clinician-services due to stigma, feelings of powerlessness, fear, and a communal ‘culture of silence’ [51].

At the clinical level, our findings urge practitioners to be especially sensitive to the mental health needs of those who have experienced violence and who would likely benefit from screening for depression. Furthermore, those that facilitate psychotherapeutic interventions should be mindful that patients they encounter with severe depression may have undisclosed experiences of violence affecting their mental health. Given the consistent depression associations with various forms of sexual violence and IPV, mental healthcare services should meet the specific needs of those experiencing prior or ongoing violence, such as ensuring privacy and safety, as well as working collaboratively with specific social services addressing sexual violence or IPV, particularly in women. At the policy-level, our findings suggest much more is needed to improve mental healthcare access and engagement in communities with high levels of violence, and further highlights that wider societal developments which could reduce interpersonal violence, might also decrease the burden of depression in LMICs.

## Conclusions

There is a strong association between an experience of violence, and current depression, current severe depression, and undiagnosed and/or untreated depression in the Brazilian population. Addressing the mental health needs of individuals and communities in LMICs affected by interpersonal violence remains a priority for policy-makers.

## Supporting information

S1 Table A list of questions, translated into English, which were used in the National Health Survey 2019 to assess violence and depression outcomes. PHQ-9: Patient Health Questionnaire.
(DOCX)

S2 Table Full results of Fig 1: Prevalence of different types of depression across populations experiencing violence. 95% CI: 95% confidence intervals. PHQ-9: Patient Health Questionnaire.
(DOCX)

S3 Table Logistic regression models investigating the association between experience of violence and various depression variables in whole survey population. Other variables included in the model are: gender; age; race; region; urban or rural locality; household income group; highest educational attainment; and marital status. Statistical significance of the adjusted Odd’s Ratio (aOR) is indicated by * for p<0.05, or ** for p<0.01. OR confidence intervals (CI) are reported at the level of 95%. PHQ-9: Patient Health Questionnaire.
(DOCX)
