# Supplementary material for: Interpersonal violence and depression in Brazil: A cross-sectional analysis of the 2019 National Health Survey
Source: PLOS Glob Public Health. 2022 Dec 2;2(12):e0001207. doi: 10.1371/journal.pgph.0001207 (PMC10021715; doi:10.1371/journal.pgph.0001207)
Supplement: S1 Table — PHQ-9: Patient Health Questionnaire. (DOCX) [file pgph.0001207.s001.docx]

| **Violence Questions** | |
| --- | --- |
| Physical violence questions | Has anyone slapped you?  Has anyone pushed, held tightly, or thrown you?  Has anyone punched, kicked, or dragged you by the hair?  Has anyone strangled, choked, or burned you?  Has anyone threated or injured you with a knife, firearm, or other weapon? |
| Sexual violence questions | Has anyone touched, manipulated, kissed, or exposed you against your will?  Has anyone threated or forced you to have sex or other sexual acts against your will? |
| Threat of violence question | Has anyone threatened to hurt you or anyone important to you? |
| **Depression Questions** | |
| Clinician depression diagnosis | Has a doctor or mental health professional (such as a psychiatrist or psychologist) given you a diagnosis of depression? |
| Depression treatments | Because of depression do you take medication?  Because of depression do you have psychotherapy? |
| PHQ-9 questions (0 points for selecting ‘no days’, 1 point for ‘less than half the days’, 2 points for ‘more than half the days’, and 3 points for ‘almost every day’) | In the past two weeks, how often have you had sleep problems, such as difficulty falling asleep, waking up frequently at night or sleeping more than usual?  In the past two weeks, how often did you have problems with not feeling rested and willing during the day, feeling tired, or without energy?  In the past two weeks, how often have you had little interest or no pleasure in doing things?  In the past two weeks, how often have you had trouble concentrating on your usual activities?  In the past two weeks, how often have you had problems with eating, such as lack of appetite or eating much more than usual?  In the last two weeks, how often were you slow to move or speak, or on the contrary, were you very agitated or restless?  In the past two weeks, how often have you felt depressed, “down” or out of perspective?  In the past two weeks, how often have you felt bad about yourself, feeling like a failure, or feeling like you've let your family down?  In the past two weeks, how often have you thought about hurting yourself in any way or thought it would be better to be dead? |
| Clinician diagnosed depression subgroup questions | Because of depression do you take medication?  Because of depression do you have psychotherapy?  Because of depression do you do acupuncture, medicinal plants and herbal medicine, homeopathy, meditation, yoga, tai chi, or some other integrative or complementary practice? (alternative medicine)  Do you regularly go to the doctor/health service for depression, or only when you have a problem?” (regular follow-up)  In any of the consultations for depression, was there a referral for follow-up with a mental health professional, such as a psychiatrist or psychologist?” (specialist referral)  Were you able to go to the appointments with a specialised mental health professional?” (specialist appointment non-attendance)  In general, to what degree does depression limit your usual activities (such as working, doing housework, etc.)? (severe limitation on activities of daily living, if respondent selected “intensely” or “very intensely”)  ‘Emergency room attendance in the last year due to depression’ was constructed from the following two questions: “when was the last time you received medical care for depression?” and “the last time you received medical care for depression, where were you treated?” |

**S1 Table:** A list of questions, translated into English, which were used in the National Health Survey 2019 to assess violence and depression outcomes. PHQ-9: Patient Health Questionnaire.
