# Supplementary material for: Interpersonal violence and depression in Brazil: A cross-sectional analysis of the 2019 National Health Survey
Source: PLOS Glob Public Health. 2022 Dec 2;2(12):e0001207. doi: 10.1371/journal.pgph.0001207 (PMC10021715; doi:10.1371/journal.pgph.0001207)
Supplement: S2 Table — 95% CI: 95% confidence intervals. PHQ-9: Patient Health Questionnaire. (DOCX) [file pgph.0001207.s002.docx]

**S2 Table – Full results of Fig 1: prevalence of different types of depression across populations experiencing violence**

|  | No Violence | | | Physical Violence Only | | | Sexual Violence Only | | | Physical & Sexual Violence | | | Threat of Violence Only | | |
| --- | --- | --- | --- | --- | --- | --- | --- | --- | --- | --- | --- | --- | --- | --- | --- |
|  | Number of Respondents | Prevalence (%) | 95% CI (%) | Number of Respondents | Prevalence (%) | 95% CI (%) | Number of Respondents | Prevalence (%) | 95% CI (%) | Number of Respondents | Prevalence (%) | 95% CI (%) | Number of Respondents | Prevalence (%) | 95% CI (%) |
| Clinician-diagnosed Depression | 7,003 | 9.5 | 9.1-9.9 | 514 | 17.8 | 15.3-20.5 | 79 | 18.8 | 12.8-26.8 | 86 | 32.1 | 23.3-42.4 | 560 | 19.4 | 17.1-21.9 |
| Current PHQ-9-detected Depression | 7,399 | 9.3 | 8.9-9.7 | 823 | 27.5 | 23.9-31.4 | 117 | 33.2 | 24.1-43.7 | 126 | 41.6 | 32.4-51.5 | 787 | 26.8 | 24.1-29.8 |
| Current Severe Depression based on PHQ-9 | 956 | 1.2 | 1.1-1.4 | 165 | 5.9 | 4.4-7.9 | 21 | 4.2 | 2.3-7.3 | 41 | 9.7 | 5.9-15.7 | 154 | 4.4 | 3.4-5.6 |
| Current Undiagnosed Depression | 4,960 | 6.0 | 5.7-6.3 | 531 | 16.1 | 13.8-18.7 | 67 | 19.9 | 13.1-29.0 | 65 | 21.8 | 15.3-30.1 | 489 | 16.2 | 13.9-18.8 |
| Current Untreated Depression | 804 | 1.0 | 0.9%-1.2 | 126 | 4.4 | 3.3-6.0 | 12 | 5.9 | 2.5-13.1 | 24 | 8.8 | 4.0-18.3 | 114 | 3.7 | 2.8-5.0 |

*95% CI: 95% confidence intervals. PHQ-9: Patient Health Questionnaire.*
