# Supplementary material for: Interpersonal violence and depression in Brazil: A cross-sectional analysis of the 2019 National Health Survey
Source: PLOS Glob Public Health. 2022 Dec 2;2(12):e0001207. doi: 10.1371/journal.pgph.0001207 (PMC10021715; doi:10.1371/journal.pgph.0001207)
Supplement: S3 Table — Other variables included in the model are: gender; age; race; region; urban or rural locality; household income group; highest educational attainment; and marital status. Statistical significance of the adjusted Odd’s Ratio (aOR) is indicated by * for p<0.05, or ** for p<0.01. OR confidence intervals (CI) are reported at the level of 95%. PHQ-9: Patient Health Questionnaire. (DOCX) [file pgph.0001207.s003.docx]

|  | | Clinician-diagnosed Depression | | Current PHQ-9-detected Depression | | Current Severe Depression based on PHQ-9 | | Current Undiagnosed Depression | | Current Untreated Depression | |
| --- | --- | --- | --- | --- | --- | --- | --- | --- | --- | --- | --- |
|  |  | aOR | CI | aOR | CI | aOR | CI | aOR | CI | aOR | CI |
| Violence Group | **No Violence** | 1 (Ref) | - | 1 (Ref) | - | 1 (Ref) | - | 1 (Ref) | - | 1 (Ref) | - |
|  | **Physical Violence Only** | 2.54** | 2.11-3.06 | 3.75** | 3.06-4.59 | 4.83** | 3.48-6.70 | 2.84** | 2.32-3.47 | 4.31** | 3.01-6.19 |
|  | **Sexual Violence Only** | 2.06* | 1.13-3.76 | 4.04** | 2.27-7.21 | 2.70* | 1.21-6.00 | 3.20** | 1.81-5.67 | 4.60** | 1.79-11.6 |
|  | **Physical & Sexual Violence** | 5.86** | 3.72-9.23 | 6.39** | 4.21-9.70 | 7.79** | 4.21-14.4 | 3.65** | 2.35-5.67 | 8.06** | 3.44-18.9 |
|  | **Threat of Violence Only** | 2.50** | 2.13-2.93 | 3.49** | 3.00-4.07 | 3.39** | 2.55-4.52 | 2.85** | 2.36-3.45 | 3.43** | 2.46-4.79 |
| Sex | **Male** | 1 (Ref) | - | 1 (Ref) | - | 1 (Ref) | - | 1 (Ref) | - | 1 (Ref) | - |
|  | **Female** | 3.10** | 2.82- 3.43 | 2.62** | 2.39-2.88 | 2.46** | 1.94-3.10 | 2.20** | 1.98-2.46 | 2.70** | 1.97-3.71 |
| Age | **15-24** | 1 (Ref) | - | 1 (Ref) | - | 1 (Ref) | - | 1 (Ref) | - | 1 (Ref) | - |
|  | **25-34** | 1.21 | 0.97- 1.52 | 0.93 | 0.79-1.10 | 1.14 | 0.72-1.80 | 0.87 | 0.73-1.05 | 1.16 | 0.68-2.00 |
|  | **35-44** | 1.88** | 1.52-2.33 | 1.02 | 0.86-1.21 | 1.28 | 0.82-2.01 | 0.85 | 0.70-1.03 | 1.64 | 0.99-2.73 |
|  | **45-54** | 2.19** | 1.77-2.71 | 1.16 | 0.97-1.38 | 1.43 | 0.89-2.28 | 0.88 | 0.72-1.08 | 1.48 | 0.89-2.46 |
|  | **55-64** | 2.24** | 1.81-2.79 | 1.12 | 0.93-1.35 | 1.61 | 1.00-2.61 | 0.83 | 0.67-1.04 | 1.23 | 0.73-2.05 |
|  | **65-74** | 1.85** | 1.45-2.36 | 0.95 | 0.77-1.17 | 1.20 | 0.69-2.09 | 0.82 | 0.65-1.04 | 0.83 | 0.47-1.48 |
|  | **75+** | 1.47* | 1.14-190 | 1.11 | 0.88-1.39 | 1.36 | 0.77-2.41 | 1.13 | 0.88-1.46 | 0.91 | 0.49-1.69 |
| Race | **White** | 1 (Ref) | - | 1 (Ref) | - | 1 (Ref) | - | 1 (Ref) | - | 1 (Ref) | - |
|  | **Black** | 0.74** | 0.64-0.86 | 0.99 | 0.87-1.13 | 1.14 | 0.83-1.57 | 1.10 | 0.95-1.28 | 1.01 | 0.72-1.42 |
|  | **Mixed** | 0.85** | 0.77-0.94 | 0.96 | 0.87-1.05 | 0.87 | 0.67-1.13 | 0.99 | 0.88-1.11 | 0.92 | 0.71-1.20 |
|  | **Asian & Indigenous** | 0.69* | 0.49-0.96 | 0.98 | 0.65-1.50 | 0.41* | 0.20-0.81 | 1.21 | 0.72-2.05 | 0.59 | 0.25-1.36 |
| Region | **North** | 1 (Ref) | - | 1 (Ref) | - | 1 (Ref) | - | 1 (Ref) | - | 1 (Ref) | - |
|  | **Northeast** | 1.34** | 1.16-1.56 | 1.30** | 1.16-1.49 | 0.99 | 0.76-1.30 | 1.15* | 1.02-1.30 | 1.12 | 0.82-1.52 |
|  | **Central-West** | 2.07** | 1.74-2.47 | 1.50** | 1.30-1.73 | 1.48* | 1.06-2.05 | 1.17* | 1.00-1.37 | 2.06** | 1.36-3.12 |
|  | **Southeast** | 2.19** | 1.87-2.57 | 1.52** | 1.33-1.73 | 1.57** | 1.14-2.16 | 1.10 | 0.95-1.27 | 1.93** | 1.32-2.82 |
|  | **South** | 3.02** | 2.56-3.58 | 1.39** | 1.19-1.61 | 1.36 | 0.98-1.91 | 0.90 | 0.76-1.08 | 1.87** | 1.25-2.80 |
| Area | **Urban** | 1 (Ref) | - | 1 (Ref) | - | 1 (Ref) | - | 1 (Ref) | - | 1 (Ref) | - |
|  | **Rural** | 0.90* | 0.81-1.00 | 0.63** | 0.57-0.71 | 0.55** | 0.43-0.71 | 0.60** | 0.53-0.67 | 0.84 | 0.64-1.10 |
| Education | **None/Incomplete Elementary** | 1 (Ref) | - | 1 (Ref) | - | 1 (Ref) | - | 1 (Ref) | - | 1 (Ref) | - |
|  | **Elementary/Incomplete High** | 0.92 | 0.80-1.07 | 0.89 | 0.77-1.01 | 0.90 | 0.67-1.20 | 0.83 | 0.72-0.97 | 1.02 | 0.72-1.43 |
|  | **High/Incomplete Higher** | 0.87* | 0.78-0.98 | 0.76** | 0.67-0.85 | 0.67** | 0.50-0.88 | 0.75** | 0.66-0.85 | 0.93 | 0.69-1.24 |
|  | **Graduate** | 0.96 | 0.83-1.12 | 0.75** | 0.65-0.88 | 0.63* | 0.40-1.00 | 0.71** | 0.57-0.87 | 0.95 | 0.62-1.45 |
| Monthly Income | **Up to 0.5x MW** | 1 (Ref) | - | 1 (Ref) | - | 1 (Ref) | - | 1 (Ref) | - | 1 (Ref) | - |
|  | **0.51-1x MW** | 0.99 | 0.87-1.11 | 0.88* | 0.79-0.98 | 0.78 | 0.60-1.01 | 0.89 | 0.78-1.00 | 0.87 | 0.61-1.25 |
|  | **1.01-2x MW** | 0.96 | 0.85-1.10 | 0.77** | 0.68-0.87 | 0.66** | 0.48-0.90 | 0.78** | 0.67-0.90 | 0.92 | 0.61-1.37 |
|  | **>2x MW** | 1.13 | 0.96-1.33 | 0.65** | 0.55-0.67 | 0.51* | 0.30-0.87 | 0.58** | 0.48-0.71 | 0.72 | 0.44-1.19 |
| Marital Status | **Married** | 1 (Ref) | - | 1 (Ref) | - | 1 (Ref) | - | 1 (Ref) | - | 1 (Ref) | - |
|  | **Separated or widowed** | 1.26** | 1.13-1.40 | 1.21** | 1.09-1.35 | 1.35* | 1.04-1.77 | 1.01 | 0.89-1.15 | 1.48* | 1.09-2.00 |
|  | **Single** | 1.01 | 0.91-1.12 | 1.09 | 0.99-1.20 | 1.22 | 0.94-1.58 | 1.04 | 0.93-1.16 | 1.31* | 1.01-1.71 |

**S3 Table –** Logistic regression models investigating the association between experience of violence and various depression variables in whole survey population. Other variables included in the model are: gender; age; race; region; urban or rural locality; household income group; highest educational attainment; and marital status. Statistical significance of the adjusted Odd’s Ratio (aOR) is indicated by * for p<0.05, or ** for p<0.01. OR confidence intervals (CI) are reported at the level of 95%. PHQ-9: Patient Health Questionnaire.
